# ADDRESSING SOCIAL ISOLATION AND LONELINESS AMONG LOW-INCOME SENIORS IN SAN FRANCISCO

**DOI:** 10.1093/geroni/igz038.2235

**Published:** 2019-11-08

**Authors:** Catherine Spensley

**Affiliations:** Felton Institute, San Francisco, California, United States

## Abstract

Felton Institute has been working to eliminate isolation and loneliness in San Francisco since its inception as a community social service agency 130 years ago. Felton offers culturally and linguistically appropriate programs and services that foster community and social connections among socially isolated, low income older adults, including those living with serious mental illness and those residing in local long-term care facilities. Examples include workforce programs for older adults; the foster grandparent program; and the senior companion program which includes friendly visiting, senior counseling, and peer escort services. This session will highlight lessons learned from these successful programs as well as how they have led to the development of new initiatives focused on conducting outreach, providing trauma-informed services, offering wellness classes, and organizing meaningful activities including a choir, intergenerational gardening, arts and dance classes, and cultural exchange opportunities to isolated older adults living in poverty in San Francisco’s Visitacion Valley.

